# Muscarinic Receptor PET in Neurodegeneration: Promise, Pitfalls, and Translational Priorities

**DOI:** 10.3390/medsci14030341

**Published:** 2026-06-23

**Authors:** Luca Filippi, Roberta Danieli

**Affiliations:** 1 Department of Biomedicine and Prevention, University of Rome ‘Tor Vergata’, Via Montpellier 1, 00133 Rome, Italy; 2 Nuclear Medicine Research Unit, IRCCS San Raffaele Roma, Via della Pisana 235, 00163 Rome, Italy; roberta.danieli@uniroma5.it; 3 Department of Human Sciences and Promotion of the Quality of Life, University San Raffaele, Via di Val Cannuta 247, 00166 Rome, Italy

**Keywords:** muscarinic receptors, PET, neurodegeneration, Alzheimer’s disease, cholinergic system, M_1_, M_2_, M_4_, positive allosteric modulators

## Abstract

Positron emission tomography (PET) of muscarinic acetylcholine receptors has evolved from a receptor-mapping exercise into a potential translational tool for probing cholinergic dysfunction in neurodegenerative disease. However, the field still lacks a clear hierarchy of clinical value across receptor subtypes, tracers, and quantitative analysis strategies, including acquisition protocols and kinetic modeling approaches. In the M_2_ arena, [^18^F]FP-TZTP has been associated with higher distribution volumes in older APOE-ε4 carriers, but the biological meaning of this signal remains uncertain in the absence of longitudinal conversion data and with quantification approaches that are difficult to implement routinely. Among M_4_ ligands, [^11^C]MK-6884 is the most advanced and reproducible tracer to date, yet its clinical evidence still rests on proof-of-concept studies, mainly in moderate-to-severe Alzheimer’s disease, with important methodological limitations. In our view, muscarinic PET should now be reframed less as a stand-alone diagnostic biomarker and more as a platform for mechanistic and pharmacodynamic studies, especially for the development and monitoring of muscarinic-positive allosteric modulators. Future progress will depend on longitudinal multicenter validation, simplified quantification pipelines, and next-generation PET systems capable of capturing tracer kinetics more efficiently.

## 1. Introduction

The cholinergic system plays a central role in maintaining cognitive function and motor coordination across the lifespan. In the aging brain, progressive alterations in cholinergic neurotransmission are commonly observed and often precede or accompany the pathological features of neurodegenerative diseases [1,2]. Among the principal targets of cholinergic signaling are the muscarinic acetylcholine receptors (mAChRs), a family of five G protein–coupled receptor subtypes (M_1_–M_5_) characterized by distinct anatomical distributions and functional roles [3]. Understanding their organization and functional dynamics has gained increasing relevance, particularly as conventional biomarkers such as amyloid-β and tau proteins do not fully account for clinical heterogeneity in disorders such as Alzheimer’s disease (AD) and Parkinson’s disease (PD).

Acetylcholine (ACh), the primary neurotransmitter of the cholinergic system, regulates higher-order cognitive processes, including learning, memory, and attention, as well as autonomic and neuromuscular functions. The five muscarinic receptor subtypes differ in both regional expression and intracellular signaling: M_1_, M_3_, and M_5_ are coupled to Gq/11 proteins and generally exert excitatory effects, whereas M_2_ and M_4_ couple to Gi/o proteins and mediate inhibitory signaling [4]. Within the central nervous system, M_1_ and M_4_ receptors are particularly prominent and play key roles in synaptic plasticity, cognitive processing, and modulation of dopaminergic pathways, while M_2_ receptors contribute to feedback regulation of ACh release.

This functional and anatomical diversity underlies the broad physiological relevance of muscarinic receptors and highlights their vulnerability in neurodegenerative conditions [5]. Cholinergic deficits, especially reductions in ACh levels and receptor availability, are consistently associated with cognitive decline and may occur before significant neuronal loss, suggesting a contributory role in disease progression [5]. These observations underscore the need to move beyond static pathological markers and to investigate dynamic neurochemical systems that more directly influence brain function.

Positron emission tomography (PET) has emerged as a powerful noninvasive technique for studying such processes in vivo, enabling quantitative assessment of receptor density, ligand binding, and neurotransmitter dynamics [6,7]. Advances in radiotracer development, including subtype-selective ligands targeting M_1_, M_2_ and M_4_ receptors, have significantly improved the spatial and functional resolution of cholinergic imaging. These tools have provided new insights into region-specific receptor alterations and their relationship with clinical symptoms across neurodegenerative disorders [8]. Muscarinic receptors are key modulators of cortico-striatal and hippocampal circuits. In the striatum, cholinergic interneurons exert a strong local influence on medium spiny neurons and interact closely with dopaminergic projections, thereby shaping motor output, habit learning, reinforcement, and reward processing. This cholinergic-dopaminergic interplay is particularly relevant in the nucleus accumbens, where it contributes to reward-related behavior and motivational control. Through these circuit-level effects, muscarinic signaling participates in attention, executive function, locomotion, and cognitive flexibility, making subtype-specific imaging of potential value in disorders characterized by both cognitive and motor dysfunction [9]. In the following sections, we provide an overview of the first attempts to image muscarinic receptors in humans using PET, with particular emphasis on their potential applications in neurodegenerative disorders, while outlining the translational challenges and future steps toward clinical implementation.

## 2. PET Radioligands Targeting Muscarinic Receptors’ Subtypes

Because the orthosteric binding pocket was highly conserved across muscarinic receptor subtypes, the development of truly subtype-selective orthosteric ligands proved inherently challenging. In the context of PET imaging, high affinity alone was insufficient; an optimal radiotracer was also required to exhibit adequate blood–brain barrier penetration, reversible in vivo kinetics, low nonspecific binding, metabolic stability, and a quantifiable signal that could be modeled reliably. Consequently, the development of muscarinic PET tracers progressively shifted from classical orthosteric agonists and antagonists toward allosteric or bitopic ligands, which were considered more likely to provide improved subtype discrimination and superior in vivo imaging performance [10].

### 2.1. M_1_ Subtype

The translational landscape of mAChR-targeted imaging is currently at a critical junction, with the M_1_ subtype serving as the primary gateway for our understanding of cholinergic dysfunction [11]. Representing approximately 60% of total muscarinic expression in the CNS, the M_1_ is far more than a structural landmark; it is a fundamental mediator of synaptic plasticity and cognitive processing in the cortex and hippocampus. However, the path from radioligand synthesis to clinical utility has been complex, demonstrating that high in vitro affinity is rarely a guarantee of in vivo success.

The early phase of M_1_-PET development, characterized by agonists such as [^11^C]xanomeline and [^11^C]butylthio-1,2,5-thiadiazol-4-yl-1,2,5,6-tetrahydro-1-methylpyridine ([^11^C]butylthio-TZTP), highlights a recurring translational hurdle where preclinical promise in non-human primates fails to translate into human specificity [12]. These tracers suffered from significant off-target binding, particularly to sigma receptors, which obscured the regional heterogeneity necessary for accurate imaging. In addition, the clinical translation of labeled xanomeline, like other muscarinic agonists, was hampered by its intolerable adverse side effect profile (mainly peripheral cholinergic adverse effects, such as nausea, vomiting, salivation and sweating), presumably resulting from the already mentioned inadequate receptor subtype selectivity.

As the search for better tools evolved, the focus shifted toward allosteric modulation, yet even this more sophisticated approach met with challenges. In this context, [^11^C]GSK1034702 served as a cautionary example, as its homogeneous distribution in the human brain underscored the difficulty of achieving high sensitivity when targeting allosteric sites [13]. It was only with the emergence of bitopic binding strategies that a significant breakthrough was achieved.

**Figure 1:**
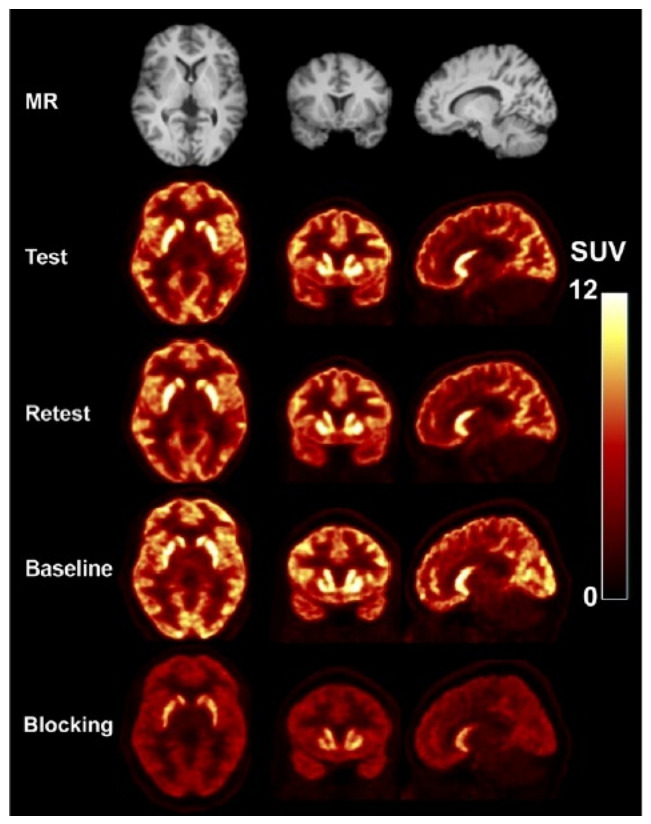
MR and corresponding co-registered PET images of ^11^C-LSN3172176 acquired under test–retest conditions in one subject and under baseline and blocking conditions in another subject. PET images were generated by summing the data collected between 30 and 60 min after injection. His research was originally published in JNM. Naganawa, M.; Nabulsi, N.; Henry, S.; Matuskey, D.; Lin, S.-F.; Slieker, L.; Schwarz, A.J.; Kant, N.; Jesudason, C.; Ruley, K.; et al. First-in-Human Assessment of11 C-LSN3172176, an M1 Muscarinic Acetylcholine Receptor PET Radiotracer. J Nucl Med 2021, 62, 553–560 [16]. © SNMMI.

By bridging the orthosteric and allosteric sites, the tracer [^11^C]LSN3172176 finally provided a robust reflection of physiological M_1_ subtype distribution, showing high test-retest reproducibility in healthy volunteers and setting a new benchmark for the field [14,15]. In a study involving healthy volunteers, Naganawa et al. successfully characterized the kinetic profile of the tracer, demonstrating high brain uptake and a regional distribution pattern (Striatum > Neocortex > Cerebellum) that aligns with known M_1_ receptor densities (Figure 1) [16]. Unlike previous M_1_-targeting candidates that suffered from poor selectivity or negligible specific binding in humans, such as [^11^C]-GSK1034702, this tracer exhibited reversible kinetics amenable to quantitative modeling. Quantitative analysis identified the 1-tissue-compartment (1TC) model as the optimal choice when arterial input functions are available, while the simplified reference tissue model 2 (SRTM2) proved to be the most robust non-invasive method using the cerebellum as a reference region. A critical finding was the differential receptor occupancy by scopolamine between striatal and non-striatal regions, likely reflecting variations in endogenous acetylcholine concentrations. With an 80-min scan duration, the tracer showed excellent test-retest reproducibility, making it a highly promising tool for quantifying M_1_ receptors in neurodegenerative and psychiatric research.

Smart et al. proposed a novel PET-based method to estimate regional ACh concentrations in the living human brain [17]. The study exploited the concept that endogenous ACh competes with radiotracers and pharmacological ligands at cholinergic receptors, thereby influencing receptor occupancy measurements. Using PET imaging with the muscarinic tracer [^11^C]LSN3172176 during scopolamine administration and the nicotinic tracer [^18^F]flubatine during nicotine challenge, the authors found that receptor blockade was consistently lower in striatal regions than in cortical and extrastriatal areas. This effect was interpreted as evidence of higher endogenous acetylcholine concentrations in the striatum, where physiological ACh competes more strongly with the administered ligands for receptor binding. Mathematical occupancy modeling enabled the generation of whole-brain maps of relative cholinergic tone. As a proof-of-concept study, the work demonstrates the feasibility of indirectly assessing in vivo neurotransmitter concentration through PET occupancy measurements.

Despite its promising characteristics, PET imaging with [^11^C]LSN3172176 has, to the best of the authors’ knowledge, thus far remained largely confined to preliminary biodistribution and methodological studies. Over recent years, research efforts have primarily focused on the optimization of quantitative imaging approaches, including kinetic modeling strategies and standardized uptake value ratio (SUVR) estimation, as well as on the evaluation of advanced technologies for head-motion correction during PET acquisition [18,19]. Additional investigations have explored non-invasive or minimally invasive alternatives to arterial blood sampling, particularly the derivation of image-based input functions from the carotid vasculature as surrogate measures for radial arterial sampling in dynamic PET protocols [20].

To date, no dedicated clinical studies investigating the application of [^11^C]LSN3172176 in patients with neurodegenerative disorders have been formally reported in the literature, or at least none have been made publicly available. This limited clinical translation may, in part, reflect the intrinsic logistical constraints associated with ^11^C radiochemistry, whose short physical half-life (~20 min) requires on-site cyclotron production and restricts widespread clinical implementation. Conversely, although several ^18^F–labeled M_1_ receptor–targeting radiotracers have been developed to overcome these limitations and to enable broader clinical applicability, none has yet achieved robust clinical validation or routine translational use [21].

### 2.2. M_2_ Subtype

Among the earliest compounds explored, AF-DX384 emerged as a promising M_2_ antagonist because of its high affinity for the receptor, although its relatively modest subtype selectivity limited its translational potential [22]. The corresponding radiotracer, [^11^C]AF-DX384, demonstrated acceptable radiosynthetic performance; however, inadequate blood–brain barrier penetration and the formation of brain-penetrant radiometabolites substantially hindered further clinical development [8]. Similar limitations have affected other early-generation compounds, including [^11^C]BIBN99, which showed improved M_2_/M_1_ selectivity but has not progressed beyond preliminary radiochemical characterization and therefore remained without meaningful biological or clinical validation [23].

In contrast, [^18^F]FP-TZTP (3-(3-(3-[^18^F]fluoropropylthio)-1,2,5-thiadiazol-4-yl)-1,2,5,6-tetrahydro-1-methylpyridine) has represented the most clinically advanced attempt at imaging M_2_ receptors in vivo [24,25]. Although structurally related to several ligands initially developed for M_1_ imaging, [^18^F]FP-TZTP displayed preferential binding toward M_2_ receptors, a feature likely attributable to slower dissociation kinetics at the M_2_ subtype. Preclinical investigations confirmed this selectivity through knockout mouse autoradiography studies, receptor-expressing cellular systems, and rat brain experiments [26]. Importantly, first-in-human PET studies demonstrated a regional distribution pattern consistent with known M_2_ receptor density across cortical, subcortical, and cerebellar structures, with tracer uptake also showing correlations with aging [27]. These findings suggest that [^18^F]FP-TZTP may serve as a potentially informative biomarker of cholinergic integrity in aging and neurodegenerative disease. A first step toward M_2_-targeted imaging applications in neurodegenerative diseases was taken by Cohen et al., who demonstrated significantly higher [^18^F]FP-TZTP distribution volumes (VT) in healthy aging APOE-ε4 carriers compared with non-carriers [28]. The comparison according to APOE genotype was chosen because the APOE-ε4 allele represents the strongest genetic risk factor for AD and is known to influence the cholinergic system, which plays a critical role in memory and cognitive function [29]. The study employed 120-min dynamic 3D PET acquisitions with attenuation correction based on 8-min transmission scans, while MRI was used for partial volume correction. Because no suitable receptor-free reference region was identified for this ligand, quantification was performed using a one-tissue compartment model with metabolite-corrected arterial plasma input data. The results showed that APOE-ε4 carriers exhibited higher VT values for the [^18^F]FP-TZTP tracer than non-carriers, a finding interpreted as reflecting a higher density of unoccupied M_2_ receptors in the brain. Importantly, these findings laid the groundwork for several clinical implications. First, elevated VT values were observed in cognitively healthy older individuals without clinical dementia, suggesting that M_2_-targeted PET imaging may enable detection of cholinergic dysfunction long before the onset of symptoms. Second, the findings suggest that the APOE-ε4 allele exerts a direct detrimental effect on the cholinergic system during aging. Finally, the reported results may help explain why older individuals and patients with AD are more sensitive to anticholinergic drugs, which block these receptors, since their cholinergic system may already be compromised or abnormally compensated. Subsequently, the same research group investigated the effects of aging and APOE-ε4 genotype on the cholinergic response to pharmacological stimulation using physostigmine and [^18^F]FP-TZTP PET imaging. They demonstrated that both increasing age and the presence of the APOE-ε4 allele were associated with greater reductions in tracer distribution volumes after acetylcholinesterase inhibition, indicating altered muscarinic cholinergic function. Importantly, the absence of an interaction between age and APOE-ε4 suggested that the APOE-ε4 genotype may independently contribute to early cholinergic dysfunction prior to overt aging-related changes [30].

However, despite these encouraging findings, [^18^F]FP-TZTP PET has been investigated mainly in the context of bipolar disorders, particularly in subjects carrying the rs324650 genetic polymorphism, whereas its potential application in patients with neurodegenerative disorders has not been further explored [31].

Another compound of interest is (R,S)-[^18^F]FMeQNB, which demonstrated subnanomolar affinity for M_2_ receptors together with moderate selectivity over M_1_ receptors. Preclinical biodistribution studies showed relatively homogeneous cerebral uptake, although significant peripheral binding, particularly at the cardiac level, highlighted the persistent challenge of separating central from peripheral muscarinic signal. While these pharmacological properties are intriguing, especially considering the importance of M_2_ receptors in both cardiac autonomic regulation and central cholinergic neurotransmission, the tracer has not yet achieved substantial clinical translation [32].

### 2.3. M_4_ Subtype

Among the currently available M_4_-selective PET radioligands, [^11^C]MK-6884 has emerged as the most extensively validated tracer and, importantly, the only compound that has demonstrated successful translation from preclinical studies to human imaging applications. This radioligand was originally developed to enable the quantification of M_4_ receptor occupancy by therapeutic agents and to support the pharmacological characterization of M_4_-positive allosteric modulators (PAMs) [33]. Its favorable pharmacological profile, combined with robust in vivo imaging properties, has positioned it as a reference compound within the field of M_4_ molecular imaging.

MK-6884 was found to exhibit subnanomolar affinity (Ki = 0.19 nM) and excellent subtype selectivity, with more than 3600-fold selectivity over other muscarinic receptor subtypes. Furthermore, the tracer displayed suitable in vitro binding characteristics, with Bmax/Kd values of 14.4 and 7.8 in monkey and human brain tissues, respectively. Autoradiographic studies performed with [^3^H]MK-6884 demonstrated a distribution pattern consistent with the known anatomical localization of M_4_ receptors, showing the highest binding density in the striatum, followed by cortical and hippocampal regions, whereas negligible binding was observed in the cerebellum [33]. Interestingly, receptor binding was markedly enhanced in the presence of the orthosteric agonist carbachol, supporting the allosteric nature of the ligand and its sensitivity to receptor conformational state.

Subsequent PET investigations in rhesus monkeys demonstrated that [^11^C]MK-6884 readily crossed the blood–brain barrier and accumulated preferentially in M_4_-rich regions, particularly within the striatum, where robust binding potentials were observed. Specificity for M_4_ receptors was further confirmed through pharmacological blocking experiments using an alternative M_4_-positive allosteric modulator (PAM) [34]. These encouraging preclinical findings paved the way for the first translational human imaging studies employing [^11^C]MK-6884 in both healthy subjects and patients affected by AD [35]. The tracer demonstrated high affinity, excellent subtype selectivity, favorable brain kinetics, and good test–retest reproducibility, with PET quantification primarily based on nondisplaceable binding potential (BPND) using cerebellum as reference region and Transient Equilibrium Tissue Ratio/Simplified Reference Tissue Model kinetic models. The clinical cohort included healthy volunteers and 10 patients with moderate-to-severe AD (MMSE ≤ 20), all receiving donepezil or rivastigmine. Importantly, [^11^C]MK-6884 binding increased after donepezil administration, indicating sensitivity to endogenous acetylcholine tone in addition to receptor density. In AD, striatal BPND remained relatively preserved compared with healthy controls, whereas marked cortical reductions, particularly in the temporal cortex, were observed, suggesting degeneration of cortical cholinergic projections with relative sparing of striatal M_4_ signaling. The tracer was also successfully used for M_4_ PAM occupancy studies with MK-4710, supporting its role as a translational biomarker for M_4_-targeted drug development. Major limitations included the small AD cohort, absence of partial volume correction, and the confounding influence of chronic acetylcholinesterase inhibitor therapy on PET signal interpretation. The main findings of this first clinical study are summarized in Table 1.

More recently, additional radiolabeled analogues derived from the MK-6884 scaffold, including two ^11^C-labeled compounds and one ^18^F-labeled derivative, have been reported. Although the ^18^F-labeled analogue demonstrated preferential uptake in M4-rich brain regions in non-human primates, its relatively low overall brain penetration limited further clinical development [35]. Consequently, [^11^C]MK-6884 currently remains the most advanced and clinically relevant M4-selective PET radioligand available.

**Figure 2:**
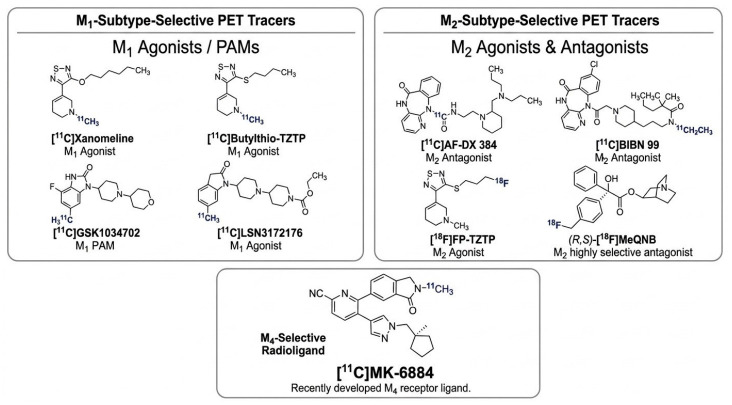
Representative radiopharmaceutical candidates targeting muscarinic receptor subtypes. The figure illustrates selected tracers proposed or investigated for M_1_ imaging (including orthosteric agonists, the positive allosteric modulator [PAM] [^11^C]GSK1034702, and the bitopic ligand [^11^C]LSN3172176, M_2_ imaging (agonists and antagonists), and M_4_ imaging (the allosteric radioligand [^11^C]MK-6884). Radionuclide labels are highlighted in blue according to the isotope used (^11^C or ^18^F).

Figure 2 summarizes the main muscarinic receptor-targeting radiotracers discussed in this section, grouped according to receptor subtype selectivity.

## 3. Translational Readiness and Potential Clinical Utility

Although some encouraging findings emerged from the first clinical applications, PET imaging remained at an early translational stage, without a clearly established role in the diagnostic or therapeutic framework of neurodegenerative diseases.

As regards M_2_-PET, the study by Cohen et al. represented an important step because it suggested that increased [^18^F]FP-TZTP binding might capture a state of cholinergic vulnerability in older individuals and APOE-ε4 carriers [28,30]. Yet the interpretation remained incomplete. A higher distribution volume in cognitively healthy participants does not by itself establish that these subjects are on a path to dementia, and the paper does not provide longitudinal evidence on conversion time or clinical trajectory. In other words, the signal may be biologically interesting, but its prognostic value is still unproven.

There is also a practical issue. Dynamic PET acquisitions, arterial input functions, and full compartmental modeling are powerful research tools, but they are cumbersome for routine clinical implementation [36]. The authors did not investigate whether qualitative or semi-quantitative image assessment could define a clinically usable positive/negative pattern, or a simple gradation of abnormality. Without this step, [^18^F]FP-TZTP remains closer to a sophisticated research tracer than to a biomarker ready for widespread use.

**Figure 3:**
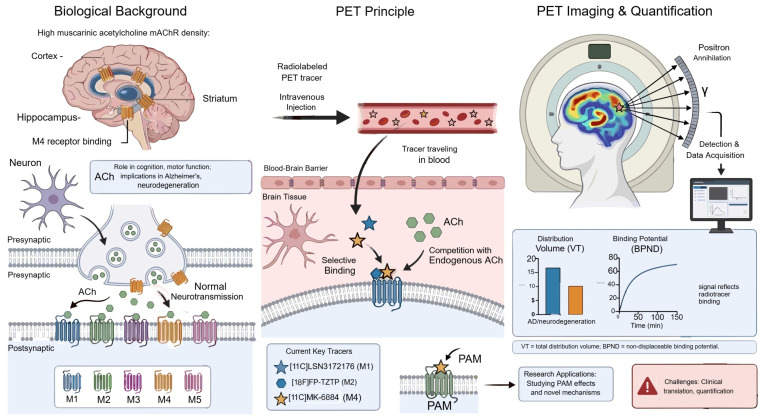
Schematic overview of muscarinic acetylcholine receptor PET imaging in neurodegeneration. The left panel shows the main CNS distribution and physiological roles of M_1_–M_5_ receptors, including striatal enrichment of M_4_ and the involvement of M_1_ in cortical and hippocampal signaling. The central panel illustrates PET tracer delivery, blood–brain barrier passage, and competition with endogenous acetylcholine, highlighting the cooperative binding mechanism at allosteric sites between imaging tracers and positive allosteric modulators (PAMs). The right panel summarizes dynamic PET quantification, including total distribution volume (VT) and non-displaceable binding potential (BPND), and highlights current translational limitations (figure was generated by authors with FigureLabs, authorized reproduction).

Among the muscarinic receptor subtypes, M_4_ emerged the most compelling target because [^11^C]MK-6884 has already demonstrated successful translation from preclinical work to human imaging [35]. Its high affinity, strong selectivity, and reproducible binding pattern make it the clearest reference compound in the field. In the first clinical studies, preserved striatal binding and reduced cortical binding in AD supported the concept that M_4_ imaging may reflect cortical cholinergic degeneration more than a global muscarinic loss. Table 2 provides a brief translational appraisal of the translational potential of the main muscarinic PET tracers, while a schematic overview of muscarin-targeted PET imaging principles and hurdles is provided by Figure 3.

Even so, this promise should not be overstated. The clinical literature is still restricted to a small number of subjects, often with moderate-to-severe disease, and the interpretation of the PET signal is complicated by chronic acetylcholinesterase inhibitor therapy, lack of partial volume correction, and the absence of large prospective validation across the full Alzheimer’s disease continuum. The tracer is also labeled with ^11^C, which limits dissemination beyond centers with cyclotron access. Unless a structurally optimized ^18^F-labeled analogue becomes available, M_4_ imaging is likely to remain largely confined to specialized research settings.

A further conceptual issue is biomarker positioning. The regional hypometabolic pattern suggested by muscarinic PET may partly overlap with PET with [^18^F]-fluorodeoxyglucose ([^18^F]FDG), which is already used as an indirect readout of synaptic dysfunction. This raises an important question: what does M_4_-directed imaging add beyond [^18^F]FDG? The answer may lie in specificity and pharmacology rather than in a simple topographic resemblance. In parallel, its relationship to newer synaptic markers such as SV2A PET also needs to be clarified, because muscarinic imaging should be integrated into the broader neurodegenerative biomarker ecosystem rather than developed in isolation [37,38]. An equally important issue concerns the clinical positioning of muscarinic PET within the current diagnostic and therapeutic framework of neurodegenerative diseases. At present, no muscarinic receptor PET tracer has been validated as a biomarker capable of guiding routine therapeutic decisions, and a positive scan cannot be interpreted as an indication for a specific treatment or management strategy. Unlike established biomarkers that are increasingly being incorporated into clinical workflows, the relationship between muscarinic PET findings and actionable clinical interventions remains largely undefined. In the near term, the greatest value of muscarinic PET is therefore likely to reside in drug development rather than routine patient care. In particular, these tracers may provide objective measures of target engagement, support dose-selection strategies, and serve as pharmacodynamic readouts for therapies aimed at modulating cholinergic signaling. Furthermore, muscarinic PET could facilitate patient stratification and trial enrichment by identifying individuals with specific patterns of cholinergic dysfunction who may be more likely to benefit from investigational treatments. Demonstrating that muscarinic PET findings can meaningfully influence therapeutic choices and improve patient outcomes will be a critical prerequisite for broader clinical adoption. The critical open questions needing clarification through future studies are summarized in Table 3.

The most attractive future use of muscarinic PET might be prospected in drug development, especially for the in vivo characterization of muscarinic-positive allosteric modulators. In this setting, the tracer is not merely a map of receptor density, but a tool for demonstrating target engagement, comparing doses, and monitoring treatment effects over time. This is where the field can become genuinely translational.

To move in that direction, future studies should be multicenter, prospective, and longitudinal, with harmonized acquisition and analysis protocols. New technologies such as long axial field-of-view PET scanners or dedicated systems like Neuroexplorer could be particularly helpful, because they may improve sensitivity, reduce scan burden, and facilitate wider implementation of dynamic protocols [20,39]. In parallel, it will be important to define simplified quantitative metrics that can eventually coexist with more sophisticated kinetic models.

## 4. Conclusions

Muscarinic PET is conceptually strong and biologically appealing, but it is still uneven across tracers and far from routine clinical adoption. At present, its greatest value is likely to be pharmacodynamic and mechanistic, not diagnostic. If the field can move beyond proof-of-concept studies and toward standardized multicenter validation, muscarinic imaging may become an important bridge between neurochemical pathophysiology and therapeutic development.

## Figures and Tables

**Table 1:** Summary of the first clinical study on M_4_-PET in Alzheimer’s Disease.

Parameter	Findings
Tracer	[^11^C]MK-6884
Target	M4 muscarinic receptor allosteric site
Affinity	Ki = 0.19 nM
Quantification	BPND using TE-TR and SRTM models
Reference region	Cerebellum
Healthy elderly cohort	7 subjects (55–85 years)
AD cohort	10 patients with moderate-to-severe AD
AD treatment status	All on donepezil or rivastigmine
Main AD pattern	Preserved striatal binding with reduced cortical binding
Most affected cortical region	Temporal cortex
Donepezil effect	Increased striatal BPND (~23%)
Occupancy application	MK-4710 target engagement studies
Major limitations	Small sample size, no partial volume correction, AChEI confounding

AD, Alzheimer’s disease; AChEI, acetylcholinesterase inhibitor; BPND, non-displaceable binding potential; Ki, inhibitory constant; SRTM, simplified reference tissue model; TE-TR, two-tissue compartment reference tissue model.

**Table 2:** Translational appraisal of the main muscarinic PET tracers.

Tracer	Subtype/Binding Profile	Reported Affinity/Selectivity	Main Strength	Main Limitation	Current Translational Position
[^11^C]LSN3172176	M_1_ bitopic ligand	High M_1_ selectivity; exact Ki not stated here	Best human-relevant M_1_ quantification to date; reversible kinetics and strong reproducibility	^11^C short half-life; clinical validation still sparse	Promising research tracer, not yet a routine clinical biomarker
[^18^F]FP-TZTP	M_2_-preferring tracer	Preferential M_2_ binding; clinical studies suggest sensitivity to cholinergic vulnerability, but subtype discrimination is less robust than for M_4_ tracers	First clinically advanced M_2_-targeting PET ligand; useful for probing age- and APOE-related cholinergic change	Dynamic/arterial modeling is demanding; prognostic meaning remains uncertain	Interesting vulnerability marker, but still needs longitudinal validation
[^11^C]MK-6884	M_4_ allosteric tracer	Ki = 0.19 nM; >3600-fold selectivity over other muscarinic subtypes	Most advanced M_4_ tracer; useful for receptor occupancy and target-engagement studies	Small studies, no broad disease validation, and ^11^C short half-life	Best current candidate for translational M_4_ imaging

APOE, apolipoprotein E; Ki, inhibitory constant; M_1_, muscarinic acetylcholine receptor subtype 1; M_2_, muscarinic acetylcholine receptor subtype 2; M_4_, muscarinic acetylcholine receptor subtype 4; PET, positron emission tomography.

**Table 3:** Key questions that should guide future prospective studies.

Open Question	Why It Matters	Preferred Study Design/Readout
Does elevated [^18^F]FP-TZTP VT predict future cognitive decline?	This would determine whether the signal is a vulnerability marker or a true prognostic biomarker.	Longitudinal cohort with clinical conversion endpoints and repeated PET
Can muscarinic PET be simplified for routine use?	Clinical adoption requires approaches that are less invasive than full dynamic modeling.	Comparison of compartmental modeling, SUVR-like metrics, and visual/semi-quantitative reads
How does muscarinic PET compare with [^18^F]FDG and SV2A imaging?	Biomarker positioning is essential to avoid redundancy and clarify complementary value.	Head-to-head multimodal studies in the same participants
Can new scanners make multicenter studies feasible?	Technical scalability will determine whether the method remains niche or becomes translatable.	Prospective multicenter protocols using long axial FOV or dedicated brain PET systems
Can M_4_ PET support PAM development and monitoring?	This is probably the most actionable near-term application.	Dose-finding and target-engagement studies with pharmacokinetic/pharmacodynamic modeling

PET: positron emission tomography; VT: volume of distribution; SUVR: standardized uptake value ratio; FDG: fluorodeoxyglucose; SV2A: synaptic vesicle glycoprotein 2A; FOV: field of view; PAM: positive allosteric modulator.

## Data Availability

No new data were created or analyzed in this study.
